# Effects of Kangaroo Care on the development of oral skills and achievement of exclusive oral feeding in preterm infants

**DOI:** 10.1590/2317-1782/20232022070

**Published:** 2023-08-04

**Authors:** Carla Ribeiro Ciochetto, Geovana de Paula Bolzan, Daniela da Silva Gonçalves, Francine Pimentel Höher da Silveira, Angela Regina Maciel Weinmann

**Affiliations:** 1 Programa de Pós-Graduação em Distúrbios da Comunicação Humana, Faculdade de Fonoaudiologia, Universidade Federal de Santa Maria - UFSM - Santa Maria (RS), Brasil.; 2 Departamento de Fonoaudiologia, Universidade Federal de Santa Maria - UFSM - Santa Maria (RS), Brasil.; 3 Hospital Universitário de Santa Maria, Universidade Federal de Santa Maria - UFSM - Santa Maria (RS), Brasil.

**Keywords:** Kangaroo Mother Care, Neonate, Preterm, Sucking Behavior, Neonatal Intensive Care, Método Canguru, Recém-Nascido, Prematuro, Comportamento de Sucção, Terapia Intensiva Neonatal

## Abstract

**Purpose:**

analyze the effects of hospitalization in the Kangaroo Neonatal Intermediate Care Unit (UCINCa), the second stage of the Kangaroo Care (KC), on the development of oral feeding skills in preterm neonates.

**Methods:**

an analytical observational study of the prospective longitudinal type, carried out in a public hospital in Southern Brazil, where infants were accompanied until hospital discharge. The sample consisted of 20 preterm neonates hospitalized at the UCINCa and 26 preterm neonates at the Conventional Neonatal Intermediate Care Unit (UCINCo), that were periodically evaluated through the levels of oral skill, in a bottle, according to the criteria of proficiency and milk transfer rate. The outcomes considered were a progression of the oral skill level, days of transition to obtain the full oral route, and days of hospital stay.

**Results:**

the duration of transition to exclusive oral feeding was shorter for preterm neonates at the UCINCa (4.5 vs. 10 days) relative to those at the UCINCo (p = 0.041). By the third assessment, all preterm neonates at the UCINCa had reached level 4, while participants at the UCINCo only achieved this level of performance on the fifth assessment. The average number of days of hospitalization was four days shorter in UCINCa participants (p=0.098).

**Conclusion:**

the admission to the UCINCa had been a further acceleration in the maturation of oral skills, which allowed for a faster transition to exclusive oral feeding as compared to neonates admitted in UCINCo.

## RESUMO

**Objetivo:**

verificar os efeitos da internação em Unidade de Cuidado Intermediário Neonatal Canguru (UCINCa), segunda etapa do Método Canguru, no desenvolvimento das habilidades de alimentação oral em recém nascidos pré-termo (RNPT).

**Método:**

estudo observacional analítico do tipo longitudinal prospectivo, realizado em um hospital público no Sul do Brasil, com acompanhamento até a alta hospitalar. A amostra foi constituída por 20 RNPT internados na UCINCa e 26 RNPT na Unidade de Cuidado Intermediário Neonatal Convencional (UCINCo), que foram avaliados periodicamente por meio dos níveis de habilidade oral, em mamadeira, conforme os critérios de proficiência e taxa de transferência de leite. Os desfechos considerados foram progressão do nível de habilidade oral, dias de transição para obtenção da via oral plena, dias de internação hospitalar.

**Resultados:**

o tempo de transição para obtenção da via oral exclusiva foi menor nos RNPT da UCINCa (4,5 dias *versus* 10 dias) quando comparados aos da UCINCo (p=0,041). Na terceira avaliação de habilidade oral, a totalidade de RNPT da UCINCa atingiu o nível 4, já os participantes da UCINCo somente alcançaram esse nível na quinta avaliação. A mediana dos dias de internação foi de quatro dias menor nos participantes da UCINCa (p=0,098).

**Conclusão:**

a internação na UCINCa favoreceu uma aceleração no processo de maturação das habilidades orais, influenciando positivamente na transição alimentar oral do RNPT, na comparação com os RNPT internados na UCINCo.

## INTRODUCTION

Kangaroo care (KC) emerged in Colombia as a way to improve the care of low birth weight newborns by promoting early skin-to-skin contact between mothers and infants. In addition to promoting emotional attachment, KC improves thermal stability and development, especially in preterm neonates, significantly reducing morbidity and mortality rates^(1)^. This approach to infant care is also associated with higher rates of breastfeeding at hospital discharge and 3-month follow-up assessments relative to conventional neonatal care^(2)^. Therefore, KC is considered a natural, inexpensive, evidence-based standard of care that should be initiated as early as possible to minimize mother-child separation^(3)^.

In the year 2000, the Brazilian Ministry of Health implemented a policy for the Humanized Care of Low Birth Weight Newborns - Kangaroo Mother Care (*política de Atenção Humanizada ao Recém-Nascido de Baixo Peso - Método Canguru*; AHRNBP - MC) in the Unified Health System, an approached based on the following tenets: care for the baby and their family, respect of individuality, promotion of skin-to-skin contact (kangaroo position), and maternal involvement in child care^(4)^.

The KC in the country is recommended in three stages. The first stage begins with prenatal care, for pregnant women who need specialized care during labor and/or birth, followed by hospitalization of the newborn in the Neonatal Intensive Care Unit (NICU) or in the Conventional Neonatal Intermediate Care Unit (UCINCo). During this stage, welcoming parents and family members, support for the woman's companion during childbirth and in gestational care, and support for breastfeeding are prioritized^(4)^. The second stage is carried out at the Kangaroo Neonatal Intermediate Care Unit (UCINCa), where there is continuity of care initiated in the previous stage, but with special attention to breastfeeding, where the preterm newborn remains continuously with its mother and the kangaroo position should be held for as long as possible^(4).^ The third stage of the KC begins with hospital discharge, preterm newborns must be clinically stable and breastfeeding, requiring care to maintain thermal stability and weight gain, which can be performed in the home environment, with monitoring shared by the hospital and primary care staff^(5)^.

The full-time presence of mothers during the second stafe of KC, may represent an incentive for breastfeeding^(6)^ can reduce the oral feeding difficulties often observed in preterm neonates.This, in turn, is attributed to the improved development of stomatognathic structures and functions caused by suckling at the breast^(7)^. A recent study observed that 37% of preterm neonates born at 29 to 33 weeks’ corrected gestational age (CGA), though clinically stable at 36 weeks’ CGA, showed inadequate oral feeding, which constituted a barrier to hospital discharge^(8)^.

The acquisition of efficient oral feeding skills is a fundamental milestone, as well as a challenge for the preterm neonates, as it requires oral skills and the ability to properly coordinate sucking, swallowing and breathing functions. However, external factors such as the possibility of suckling at the breast, the hospital environment, and the approach to offering food can either facilitate or hinder these abilities^(9)^. Thus, the present study aimed to verify the effects of hospitalization in UCINCa, second stage of the KC, on the development of oral feeding skills in preterm neonates.

## METHODS

### Study site, design, and ethical considerations

This was a longitudinal analytical observational study performed in the NICU of a public hospital in Southern Brazil, during the second stage of KC. The unit has 25 beds: 10 for high-risk patients, 10 for conventional intermediate care, and 5 for KC. Data were collected between April 2018 and January 2019. This study was approved by the Research Ethics Committee of the institution where it was conducted under protocol number 183.559, CAAE 11155312.7.0000.5346. All subjects provided written consent for participation.

### Sample selection procedures

Had been done a sample size calculation, considering a significance level of 5%, a power of 70% and a sampling error of 30%. The estimated sample size was 26 preterm neonates in each study unit, UCINCa and UCINCo.

Initially, the medical records of preterm neonates were pre-screened based on the following inclusion criteria: ≤34 weeks gestational age at birth and clinical stability to begin oral feeding. Other inclusion criteria were the absence of: head or neck malformations, cardiac abnormalities or genetic syndromes; maternal or neonatal conditions that contraindicated KC, such as galactosemia; grade III or IV intracranial hemorrhage; bilirubin encephalopathy; bronchopulmonary dysplasia; 1- and 5-minute Apgar scores <7 and twin birth.

### Participants

Neonates were first assessed upon receiving medical recommendation to initiate oral feeding, which in turn occurred upon discharge from high-risk care. At this point, neonates who had clinical stability were allocated to either the UCINCa or UCINCo.

The allocation in each unit occurred according to the operational criteria of the hospital’s Neonatal Care Service. To participate in the UCINCa, the mother must have the maternal desire to participate, availability of time, social support network, knowledge and ability to handle the baby in the kangaroo position, ability to recognize the signs of stress and risk situations for the newborn^(4)^. If these criteria were not met, infants were allocated to the UCINCo_._

### Data collection

The following information was collected from electronic records at the hospital: mother’s age and education, primiparity vs multiparity, work during pregnancy, type of delivery, gestational age and weight at birth, 1- and 5-minute Apgar scores, days of life, gestational age and volume of breastmilk (ml/kg/day) prescribed at the initiation of oral feeding.

Intra-uterine growth curves developed by the INTERGROWTH-21^st^Project were used to classify birthweight for gestational age^(10,11)^. Neonates were classified as small-for-gestational-age (SGA) when birthweight (g) was below the 10th percentile for gestational age and as large for gestational age (LGA) when weight was greater than or equal to the 90th percentile. All other infants were classified as adequate for gestational age (AGA).

## PROCEDURES

After medical clearance for oral feeding, neonates were assessed by a speech pathologist with experience in neonatal care. Infants were screened for signs of readiness for oral feeding and, if these were present, a first feeding session was conducted to evaluate infants’ oral skills, as proposed by Lau and Smith^(12)^. The choice for this resource had been achivied by the possibility of using a quantitative method of evaluation, which would allow monitoring the progression of preterm neonates regarding the maturation of oral skills, in addition to the initial diagnosiss.

In this assessment, milk was offered in Dr. Brown’s baby bottle with a phase 1 narrow-base nipple, which mothers were instructed to use only for the quantitative assessment of the first feeding session and all mothers were encouraged to breastfeed their children and encouraged to practice breastfeeding as well. The assessment began with the insertion of the bottle nipple into the mouth of the neonate, and sessions had a maximum duration of 20 minutes. If the infant showed any signs of fatigue or stress, the feeding session was interrupted by the examiner. Infants’ oxygen saturation and heart rate were monitored throughout the assessment of oral feeding.

From its measures of proficiency - PRO (% milk consumed in the first 5 minutes relative to the total amount prescribed) and transfer rate - TT (milk consumed as a function of time, measured in ml/minute), its possible to infer that the success in the evolution of oral feeding can be evaluated through the classify infants' oral feeding ability, which allows the classification of neonates into levels of oral feeding ability and indicates the therapeutic approaches that must be followed for each level^(12)^.

The preterm neonates, when it had achieved a PRO of less than 30% and a TT of less than 1.5 ml/min, was classified as level 1, corresponding to a low ability for oral feeding and low resistance to feeding, that is, it presented high fatigue, being recommended oral sensorimotor stimulation and resistance training. When the preterm neonates obtained a PRO of less than 30% and a TT of 1.5 ml/min or more, it was classified as level 2, presenting a low ability for oral feeding and high resistance, that is, low fatigue, being recommended to perform of oral sensorimotor stimulation. When the preterm neonates obtained a PRO greater than 30% and a TT less than 1.5 ml/min, it was classified as level 3, presenting high oral ability and high resistance, low fatigue, and resistance training was recommended. And when obtaining a PRO greater than 30% and a TT of 1.5 ml/min or more, it was classified as level 4, being classified with high ability and high resistance for the oral route, requiring no interventions^(12)^.

All preterm neonates classified in levels 1, 2, or 3 were submitted to oral sensorimotor stimulation, performed once a day, six time per week, by an experienced speech pathologist, according to the standard operating procedure of the NICU. The stimulation was also permormed in the two care units: UCINCa and UCINCo.

The oral sensorimotor stimulation involved the use of the Premature Infant Oral Motor Stimulation (PIOMI). The protocol which consists of a 5-minute intervention with 8 stages of tactile/oral manipulation performed with a gloved finger and involving the lips, cheeks, jaw, tongue, and gums followed by 2 minutes of non-nutritive sucking^(13)^. This intervention aims to elicit muscle contractions and increase muscle strength and resistance while providing orofacial sensory stimulation and an opportunity for suction training.

In order to monitor the process of oral skills maturation, the assessment of the oral skill level was repeated by the same speech pathologist, with a break period of four to six days, for all children, regardless of the hospitalization unit, until reaching level 4 of oral skill.

### Outcomes

The outcomes assessed were the progression of oral feeding skills, the length of the transition to full oral feeding, and the duration of hospitalization, both measured in days. Secondary outcomes consisted of additional measures of feeding performance, namely proficiency, transfer rate, and overall performance (% milk consumed relative to the total amount prescribed).

Days of transition from gavage to full oral feeding were counted from the time of study enrollment, that is, the introduction of oral feeding, until the achievement of independent oral feeding, or the date of removal of the feeding tube.

The length of hospitalization was measured in two ways: total days of hospitalization (from birth to discharge) and days of hospitalization from the introduction of oral feeding to hospital discharge. Weight gain was calculated as the difference in weight between the start of oral feeding and the removal of the feeding tube.

### Statistical analysis

Data were compiled and analyzed using STATA v. 10. Mean values were compared using Student’s T-test, while median values were analyzed using the Mann-Whitney U. Categorical variables were compared using Pearson’s chi-square test. Results were considered significant at p≤ 0.05.

## RESULTS

**Figure 1:**
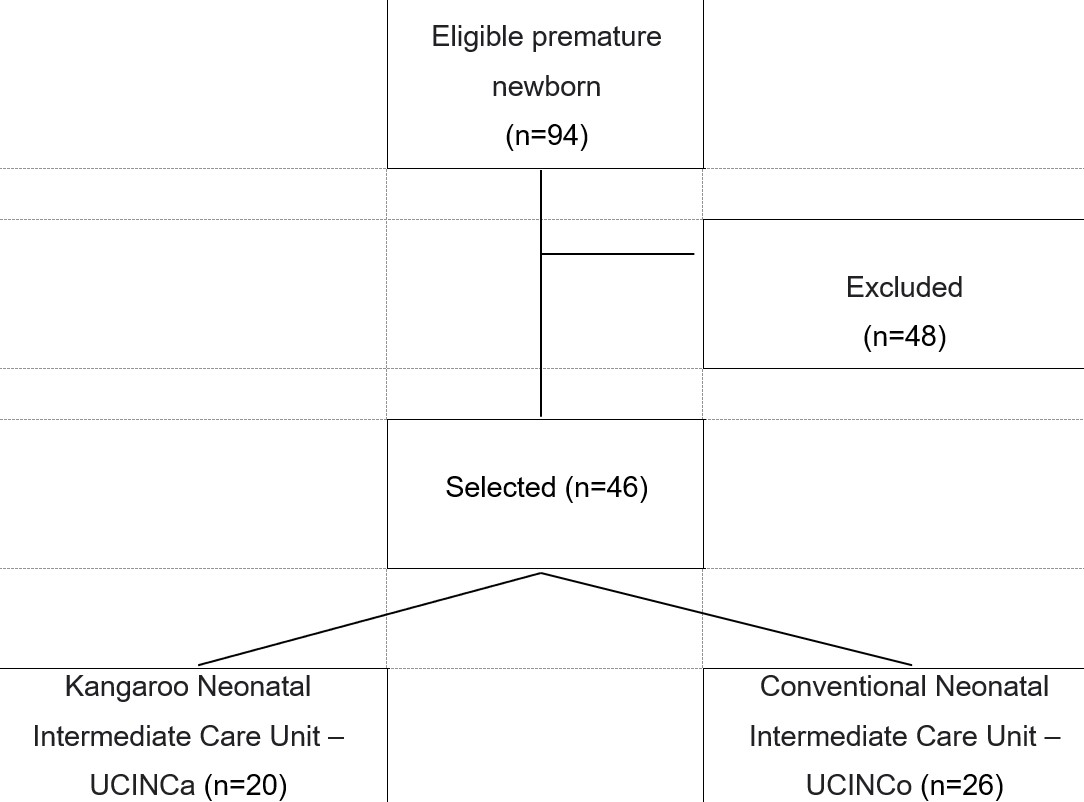
Compositon flowchart from the composition study sample

During the data collection period, 258 neonates were admitted to the NICU. Ninety-four were born at 34 weeks gestational age or less. A total of 46 neonates met the inclusion criteria for the study. Twenty were admitted to the UCINCa and 26 to the UCINCo (Figure 1).

Maternal and birth characteristics are described in Table 1.

**Table 1:** Maternal and birth characteristics of preterm neonates in each of the hospital units examined

**Variables**	**UCINCa (n=20)**		**UCINCo (n=26)**			
	**% (n)**	**Mean** ± **SD**	**% (n)**	**Mean** ± **SD**	**P- value**
**Mother**					
Age (years)		28.5 ± 7.9		27.7 ± 7.2	0.731*
Education (years)		10.9 ± 2.5		9.6 ± 3.2	0.136*
Primiparous	60 (12)		38.5 (10)		0.147**
Work during pregnancy	55 (11)		38.5 (10)		0.264**
Type of Delivery					0.482**
Vaginal	25 (5)		34.6 (9)		
Cesarean Section	75 (15)		65.4 (17)		
**Neonate**					
Male	45 (9)		53.8 (14)		0.552**
Gestational age (weeks)		32.0 ± 0.9		31.8 ± 2.1	0.357*
Weight at birth (g)		1583 ± 302		1619 ± 405	0.367*
1-Minute APGAR		7.2 ± 1.9		7.2 ± 1.8	0.494*
5-Minute APGAR		9.1 ± 0.9		8.8 ± 1.0	0.244*
Weight for gestational age					0.356**
SGA	20 (4)		15.4 (4)		
AGA	70 (14)		57.7 (15)		
LGA	10 (2)		26.9 (7)		

* Student’s T-test;

** Pearson’s Chi-square Test

**Caption**: UCINCa = Kangaroo Intermediate Care Unit; UCINCo= Conventional Intermediate Care Unit; g = grams; min = minute; SD = standard deviation; SGA = small for gestational age; AGA = adequate for gestational age; LGA = large for gestational age.

Maternal characteristics did not differ between participants admitted to the UCINCa and the UCINCo. Infant characteristics also showed no differences between groups. Although most infants in both groups were classified as having adequate weight for gestational age at birth, the percentage of neonates classified as LGA was greater in the conventional NICU.

Table 2 shows the gestational age, days of life, and volume of milk prescribed to participating infants when first cleared for oral feeding.

**Table 2:** Corrected gestational age (weeks), days of life, and volume of milk prescribed (ml/kg) on clearance for oral feeding among preterm neonates in the Kangaroo and Conventional Neonatal Intermediate Care Units

**Variables**	**UCINCa (n=20)**	**UCINCo (n=26)**	**P- value**
CGA when cleared for OF (wks) *	34.2 ± 0.9	34.4 ± 1.3	0.338
Days of life **	15 (8.5 - 22.5)	11 (5 - 26)	0.739
Volume of milk (ml/kg/day)*	144 ± 32.8	126 ± 37.7	0.047

* Student’s T-test (values expressed as mean and standard deviation);

** Mann-Whitney U test (values expressed as median, 1st, and 3rd quartiles)

**Caption**: UCINCa= Kangaroo Intermediate Care Unit; UCINCo = Conventional Intermediate Care Unit; CGA = corrected gestational age; OF = oral feeding; wks = weeks.

At the start of oral feeding, neonates at the UCINCa were at 34.2 ± 0.9 weeks CGA while those at the UCINCo were at 34.4 ± 1.3 weeks. Median postnatal age was 15 and 11 days in the UCINCa and UCINCo, respectively (p >0.05). Infants at the UCINCa were prescribed a greater volume of milk (ml/kg/day) than those at the UCINCo (p=0.047).

Table 3 demonstrates the changes in oral feeding levels in each group, as well as the results of the following feeding performance measures: proficiency, transfer rate, and overall performance.

**Table 3:** Evolution of feeding performance (proficiency, transfer rate and overall performance) and oral skill levels in neonates included in this study

**Variables**	**1^st^ Assessment**			**2^nd^ Assessment**			**3^rd^ Assessment**			**4^th^ Assessment**	**5^th^ Assessment**
	**UCINCa (n=20)**	**UCINCo (n=26)**	**P- value**	**UCINCa (n=12)**	**UCINCo (n=19)**	**P- value**	**UCINCa (n=3)**	**UCINCo (n=9)**	**P- value**	**UCINCo (n=3)**	**UCINCo (n=2)**
Weight (grams)^a^	1804 ± 226	1983 ± 453	0.056	1930 ± 295	2139 ± 420	0.071	2093 ± 253	2470 ± 437	0.097	2917 ± 395	3242.5 ± 279
PRO (%) ^a^	32.5 ± 20.3	29.8 ± 17.5	0.317	46.3 ± 26.2	25.7 ± 19.7	0.003	38.2 ± 12.5	42.9 ± 15.9	0.323	21.0 ± 3.2	47.9 ± 17.1
TR(ml/min) ^a^	1.8 ± 1.1	1.6 ± 1.1	0.194	2.5 ± 1.9	1.9 ± 1.5	0.043	2.7 ± 0.5	2.9 ± 1.4	0.400	2.2 ± 0.5	5.6 ± 1.6
OP (%) ^a^	43.9 ± 25.1	36.9 ± 21.9	0.156	56.7 ± 25.7	39.5 ± 22.0	0.028	52.0 ± 19.1	57.3 ± 31.4	0.403	67.9 ± 28.3	83.3 ± 23.6
Oral feeding skill level (%(n))^b^											
Level 1, 2 or 3	60 (12)	76.9 (20)	0.216	33.3 (4)	52.6 (10)	0.293	0 (0)	33.3 (3)	0.513	66.7 (2)	0 (0)
Level 4	40 (8)	23.1 (6)		66.7 (8)	47.4 (9)		100 (3)	66.7 (6)		33.3 (1)	100 (2)

a Student’s T-test (values expressed as mean and standard deviation);

b Pearson’s Chi-square test (percentage and frequency)

**Caption**: UCINCa= Kangaroo Intermediate Care Unit; UCINCo = Conventional Intermediate Care Unit; PRO= Proficiency; TR = Transfer rate; OP = Overall performance.

In the first assessment, no significant differences were observed between feeding performance variables in neonates from the UCINCa and UCINCo. However, on the second assessment, infants admitted to the UCINCa out performed those at the UCINCo in measures of proficiency, transfer rate, and overall performance (p=0.003, p=0.028, and p=0.043, respectively). These results were reflected in the progression of oral feeding skills, as evidenced by the increasing number of infants at the UCINCa achieving oral feeding level 4. In this group, 100% of infants had achieved level 4 by the 3^rd^ assessment as opposed to 66.7% in the UCINCo. Only infants at the UCINCo underwent a fourth and fifth assessment, and it was only on the latter that 100% of infants achieved level 4.

**Figure 2:**
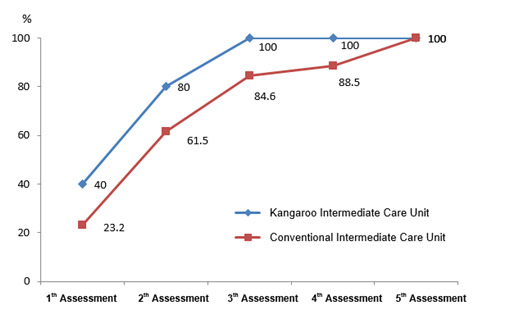
Cumulative percentage of preterm neonates who achieved oral feeding level 4 at the two hospital units examined

Figure 2 illustrates the cumulative percentage of infants who achieved oral feeding level 4 on each assessment.

On the first assessment, 40% of infants at the UCINCa and 23.1% of those at the UCINCo had reached level 4. On the second assessment, those figures increased to 80% and 61.5%, respectively, while on the third assessment all infants at the UCINCa and 84.6% of those at the UCINCo had reached oral feeding level 4. It was only on the fifth assessment that the percentage of infants at the UCINCo who reached level 4 reached 100%.

Table 4 contains information on the duration of hospitalization and transition to oral feeding, as well as weight gain during the transition from tube to independent oral feeding and the characteristics of neonates at the time of hospital discharge.

**Table 4:** Duration of the transition to full oral feeding, days of hospitalization, weight gain, and characteristics of neonates on hospital discharge at the two units

**Variables**	**UCINCa** **(n=20)**	**UCINCo** **(n=26)**	**P-value**
Days of transition to independent OF*	4.5 (3 - 9)	10 (6 - 14)	0.041
Days of hospitalization*	24 (19.5 - 37.5)	27 (19-42)	0.602
Days of hospitalization after OF*	11 (8.5 - 15)	15 (9 - 19)	0.098
Weight gain (g)**	144 ± 185	371 ± 356	0.007
Weight at tube removal (g)**	1943 ± 293	2338 ± 547	0.003
CGA at discharge (wks)*	35.7 (35.3 - 37.4)	36.5 (35.6 - 37.3)	0.102
Weight at discharge (g)**	2072 ± 250	2484 ± 558	0.002

* Mann-Whitney U Test (values expressed as median, 1st and 3rd quartiles);

** Student’s T-test (values expressed as mean and standard deviation)

**Caption**: UCINCa= Kangaroo Intermediate Care Unit; UCINCo = Conventional Intermediate Care Unit; CGA = corrected gestational age; wks = weeks; g = grams; OF = oral feeding.

The transition from tube to independent oral feeding was significantly faster for infants admitted to the UCINCa. In this group, the transition took 4.5 days as compared to 10 days in the UCINCo (median) (p = 0.041). The average days of hospitalization (total and from the start of oral feeding) was 4 days less UCINCa, however this result did not show a statistically significant difference. It is also important to note that the weight gain during the feeding transition (p = 0.007) and weight at hospital discharge (p = 0.002) was significantly lower among infants in the UCINCa relative to those in the UCINCo.

## DISCUSSION

Our aim in this study was to assess the influence of admission to a UCINCa on the development of oral skills in preterm neonates. We observed an acceleration in the maturation of these abilities in children admitted to the UCINCa as compared to the UCINCo. The full-time presence of mothers in the unit, the skin-to-skin contact, and especially the encouragement of breastfeeding allowed for a faster progression through oral feeding levels and therefore a significantly shorter transition from tube to independent oral feeding. However, weight gain was lower in UCINCa newborns.

When routine care is provided by parents inside the NICU, family bonds are strengthened, leading to a reduction in parental stress and creating a supportive infant care environment. If present, the infant’s family, and especially the mother, can help identify signs of readiness and desire for oral feeding and receive encouragement to breastfeed, resulting in a positive influence on the development of oral skills in preterm neonates^(14)^.

Breastfeeding contributes to the development of stomatognathic structures and functions. The movement of masticatory muscles contributes to the maturation process and influences bone development, improving the neuromuscular condition of oral structures. Additionally, breastfeeding helps children learn to correctly position their tongues, seal the lips, and breathe correctly during suction^(7)^. Therefore, early initiation of KC and, consequently, parental involvement in breastfeeding in preterm infants will have a positive effect on oral-motor development and accelerate the achievement of independent oral feeding^(15)^.

In the present study, findings regarding infants exposed to KC confirmed its role in the acceleration of oral skill development. Though the percentage of children with adequate oral skills (level 4) at the start of the study was higher in the UCINCa, 100% of them achieved level 4 maturity by the third assessment, while in the conventional NICU, this only occurred by the fifth assessment. This acceleration led to a reduction in the number of days required to achieve independent oral feeding. Infants in the UCINCa transitioned to full oral feeding in 4.5 days while those in conventional care required 10 days (p = 0.041).

To assist in the maturation of oral skills, it had been used an mediation with the PIOMI, regardless of the patient hospitalization unit, until the preterm newborn reached level 4 of oral skill. Studies show that this technique is effective and favors the acquisition of full oral feeding in preterm newborns^(16-22)^. A reduction in the transition time of 2.6 days^(16)^, 3.7 days^(21)^, 4 days^(22)^ and 13.5 days^(20)^ were described in preterm infants submitted to PIOMI, in comparison with the control group.

In this study, CGA did not significantly differ between the two participant groups at the start of oral feeding. According to a meta-analysis of the effects of KC on the time to start breastfeeding in low-weight preterm neonates, mothers at a UCINCa began breastfeeding an average of 2.6 days before those in a conventional care unit^(23)^. Mothers who participate in KC are thought to be in closer contact with their children and feel more secure to initiate breastfeeding. This phenomenon was not demonstrated in the present study, possibly because all participating neonates were cleared to start oral feeding on the day they were transferred to the units of study.

Though the duration of hospitalization did not differ between units, we found that the length of hospitalization from the start of oral feeding was 3 to 4 days shorter, on average, for infants in the UCINCa relative to those in the conventional care unit. Though this difference was not statistically significant, it is a highly relevant finding from a clinical practice standpoint. In a previous systematic literature review, the authors observed that preterm neonates who participated in KC had shorter hospitalizations than their peers in conventional care, with a difference of 1 day and 18 hours^(24)^. Timely hospital discharge frees hospital beds for other preterm neonates who may benefit from this type of care, thereby increasing their chances of survival. In addition, it provides the strengthening of the mother-baby relationship, especially with the arrival of the preterm neonates in ​​their own home, a natural environment for families.

When initiated early (within 24 hours of birth), the KC is associated with a statistically significant reduction in the duration of hospitalization. However, early initiation is not always possible, as it also depends on the clinical status and stability of the preterm neonates. Additionally, it is important to consider the mother’s post-partum health and ability to provide the necessary care to their baby^(2)^.

Weight gain during the food transition period, as well as weight at tube removal and at discharge, differed significantly between care groups. On the other hand, a recent study found that the length of time during which children remain in the kangaroo position also influences weight gain. Babies who remain in this position for at least 6 hours a day gain more weight than those who do so for less time, especially for less than 2 hours, which is generally the case in conventional care units. These findings reinforce the recommendation to initiate KC as soon as possible and also to maintain it for as long as possible^(25)^.

In the present study, the time of permanence in the kangaroo position was not measured, though the aforementioned findings do underscore the importance of increasing and monitoring the time spent by infants in this position to potentiate the benefits of KC. However, it must be noted that the kangaroo position is not synonymous with breastfeeding. KC constitutes a much larger set of interventions that include, in addition to skin-to-skin contact (kangaroo position), exclusive breastfeeding,and the monitoring of newborns^(26)^.

There is high-level evidence that KC is safe and low-cost intrervention, and effectively reduncion the length of hospitalization^(27)^. A multidisciplinary team must also be involved in the action plans of NICUs since actions and interventions based on the knowledge of professionals who work with breastfeeding are likely to have larger effects on infant health.

The present study shows limitations, the sample size and may be attributed to the small number of beds available for in the UCINCa. The absence of monitoring the duration of time spent by mothers and neonates in the kangaroo position during their stay at the UCINCa, which may also have influenced results, and it is unlikely that the evaluator could be blinded to the objectives of the study, due to the size of the team of speech pathologist.

However, despite these limitations, our study obtained positive results. Furthermore, the team sought to play a dynamic role in providing instructions on KC to all mothers, emphasizing that the presence of a baby bottle did not interfere with this practice, as this instrument can be used when mothers are not present or when there is a need to complement the infant’s diet in the hospital setting.

The monitoring of oral skills was important to analyze the nutritional evolution of preterm neonates in different hospitalization settings. The present findings support the increased adoption of KC in the hospital environment, encouraging parents to engage in early contact with their children and incentivizing family bonding and breastfeeding.

## CONCLUSION

The present findings demonstrated that admission to a UCINCa accelerated the maturation of oral skills in preterm neonates, facilitating the achievement of independent oral feeding. The full-time presence of mothers at the kangaroo care unit represents an important incentive to breastfeed, accelerating the evolution of oral skills and, consequently, the transition from tube to independent oral feeding.
